# Estimation of Dairy Cow Survival in the First Three Lactations for Different Culling Reasons Using the Kaplan–Meier Method

**DOI:** 10.3390/ani12151942

**Published:** 2022-07-30

**Authors:** Wilhelm Grzesiak, Krzysztof Adamczyk, Daniel Zaborski, Jerzy Wójcik

**Affiliations:** 1 Department of Ruminants Science, West Pomeranian University of Technology in Szczecin, Klemensa Janickiego 29, 71-270 Szczecin, Poland; wilhelm.grzesiak@zut.edu.pl (W.G.); jerzy.wojcik@zut.edu.pl (J.W.); 2 Department of Cattle Breeding, Institute of Animal Sciences, University of Agriculture in Krakow, Mickiewicza 24/28, 30-059 Kraków, Poland; rzadamcz@cyfronet.pl

**Keywords:** cluster analysis, Cox proportional hazards model, culling, dairy cow, Kaplan–Meier curve, survival analysis, survival table

## Abstract

**Simple Summary:**

From a breeding and production point of view, the length and quality of life of dairy cows are directly determined (more or less) by voluntary decisions made by the breeders and technical staff in human–animal–environment relationships. In this case, economic conditions are the key roles that greatly complicate the decision processes, especially when it concerns the whole herd (not solely single animals). On the other hand, increasing social pressures on the continuous improvements of animal welfare and pro-environmental agricultural practices, including high-producing dairy cows, can be seen. Therefore, the aim of the present study was to analyze survival curves for cows culled for different reasons over three successive lactations and to determine the effects of various factors on cow survival. The main culling categories were reproductive disorders—40%, udder diseases—13 to 15%, and locomotor system diseases—above 10%. The survival curves for cows from individual culling categories had similar shapes. The greatest influences on the relative culling risks were exerted by: age at first calving, lactation length, calving interval, production subindex, breeding value for longevity, temperament, and average daily milk yield. A more accurate method of determining culling reasons would be required.

**Abstract:**

The aims of the study were: (i) to compare survival curves for cows culled for different reasons over three successive lactations using the Kaplan–Meier estimator; (ii) to determine the effects of breeding documentation parameters on cow survival; (iii) to investigate the similarity between culling categories. The survival times for a subset of 347,939 Holstein-Friesian cows culled between 2017 and 2018 in Poland were expressed in months from calving to culling or the end of lactation. The survival tables were constructed for each culling category and lactation number. The survival curves were also compared. The main culling categories were reproductive disorders—40%, udder diseases—13 to 15%, and locomotor system diseases—above 10%. The survival curves for cows from individual culling categories had similar shapes. A low probability of survival curves for metabolic and digestive system diseases and respiratory diseases was observed in each of the three lactations. The contagious disease category was almost non-existent in the first lactation. The greatest influence on the relative culling risk was exerted by age at first calving, lactation length, calving interval, production subindex, breeding value for longevity, temperament, and average daily milk yield. A more accurate method of determining culling reasons would be required.

## 1. Introduction

From a breeding and production point of view, the length and quality of life of dairy cows are directly determined (more or less) by voluntary decisions made by the breeders and technical staff in human–animal–environment relationships [1]. In this case, the key roles involve macroeconomic (e.g., changes in the global relationships between milk prices and animal feeding costs as well as breeding animal prices) and microeconomic (e.g., veterinary service expenditures) conditions, which greatly complicate the decision processes, especially when it concerns the whole herd (not solely single animals) [2]. On the other hand, it can be seen that social pressure on the continuous improvements of animal welfare (promoting grazing management systems and free-stall housing instead of tie-stall housing, social recommendations, and expectations about the humane treatment of animals at each stage of their handling, as well as respecting the five freedoms as minimum obligations to animals) and pro-environmental agricultural practices (especially the reduction of carbon footprints) involving high-producing dairy cows increases [3,4,5,6,7]. These factors result in the need for an increasingly detailed analysis of the length and quality of a cow’s life, first utilizing routinely collected breeding data [8]. Among them, special attention should be paid to data on the longevity and survivability of cows and their culling reasons [9,10]. Culling decisions constitute a major challenge to dairy owners and managers. Culling has a huge impact on breeding progress but it also generates costs associated with replacement heifers that account for a substantial part of the dairy budget. The importance of culling decisions is reflected in the fact that owners of particularly large herds, who are not normally involved in the management decisions of individual cows, often directly participate in the decision-making process for selecting animals to cull [11,12]. However, the aim of the breeding work is not to completely eliminate culling, but to minimize the rate of cow culling unplanned by breeders (involuntary culling) at an increased level of voluntary culling [13,14]. Therefore, the knowledge of culling reasons and their dynamics (especially for the first three lactations of Holstein-Friesian cows) exert the greatest effects on breeding decisions as well as breeding and production results at the herd and population levels [15,16,17,18,19]. However, it should be emphasized that the outcome (animal survivability or longevity and culling) is not only associated with the single culling decision, but also with the life history of an animal, including the age of first calving and feeding intensity that affects the milk yield of cows [7,20]. A significant association between longevity and culling reasons with milk performance traits and the reproduction of cows has been indicated, taking into account management conditions at the same time (e.g., herd size, culling season). For instance, the need to consider factors, such as the percentage of Holstein-Friesian genes, the breeding values of cows and sires, age at first calving, the length of calving interval, and milk performance, has been confirmed in the evaluation of longevity and culling reasons in high-yielding animals [21,22,23,24,25,26,27]. Considering the above, the analysis of longevity and survivability of dairy cows in terms of breeding and production aspects has been a top research topic in recent years. Special attention has been paid to the selection of methods precisely determining the relationships among variables [28,29,30,31]. It appears that survival analysis is one of them. It is used for investigating the association between longevity, survivability, culling reasons, and routine herd data in terms of dairy cow culling.

The basis for survival analysis is the duration process, characterized by a certain relationship, i.e., the occurrence of two events (e.g., the initial and final ones, such as a cow’s birth and her culling) [32,33,34,35,36,37,38]. The time interval between these events is a random variable [39,40]. Such an approach allows for the development of a survival model, which is the probability distribution of this variable, assuming positive values [34]. The Kaplan–Meier estimator is one of the most frequently used methods to analyze survival data and make comparisons between groups of individuals [36,41], such as cows culled for different reasons [14,42,43]. One advantage of the survival analysis, also known as the failure time or event time analysis, is the fact that the information from cows that are not culled by the time the data recording is complete can also be retained. Thus, records from culled (uncensored) and surviving (censored) cows can be treated jointly and included in the analysis, so that all the available information is properly used [17,18,19,37,38,44,45]. Cows still alive at the end of the study period cause problems when using other methods for the survival evaluation because their actual life spans are unknown. Therefore, the survival analysis offers multiple advantages over traditional linear models in terms of better fitting the survival data and using time-dependent variables properly [14,19,42]. It is based on the so-called hazard rate, i.e., the probability of a cow being culled at time *t*, given that the cow has been alive prior to *t* [41]. The hazard rate is usually modeled as a product of a baseline hazard function (which relates to the natural aging process) and an exponential function of factors potentially affecting culling risk, such as herd–year–season, milk production level, or genetic causes (e.g., sire effect). The hazard rate can be calculated from both uncensored and censored records [17,38,44,45]. Historical data are usually used to construct survival models, although this method can also be the basis for prediction [46]. Cluster analysis, on the other hand, allows for the grouping of objects according to specific criteria, which in turn enables the creation of a useful classification system [47,48].

The main aims of the study were: (i) to describe and compare survival curves for cows culled for different reasons over three successive lactations using the Kaplan–Meier estimator; (ii) to determine the effects of breeding documentation parameters on the survival of cows, especially their influences on the relative risks in animals culled for various reasons; (iii) to investigate the similarities between individual culling categories based on survival curves and the cluster analysis.

## 2. Materials and Methods

A total of 347,939 milk-recorded Holstein-Friesian cows culled between 2017 and 2018 in Poland were included in the study. They were mostly kept under an outdoor/indoor system (i.e., grazed during the spring and summer and had access to paddocks throughout the year). A stratified sample of the original dataset was used for a detailed analysis. Table 1 presents the structure of cull cows in the original dataset and the selected subset. The cows were divided into nine culling categories according to the breeding documentation (the analysis did not include the “old age” reason), whereas the reproductive disorder category also comprised sterility (Table 1).

The survival time is expressed in months from calving to culling or the end of lactation (the analysis included three successive lactations). A variable indicating censored observations was also created (with values of 0 and 1, 1—with censored observation, i.e., those that survived the observation period and their further fate was not followed at a given moment of analysis and 0—cows with full observation, i.e., those that were culled during the observation period) [16,18,32,33,37,39,45].

For the initial analysis, survival tables (presented in Supplementary Tables S1–S3), including individual indicators in class intervals were constructed for each culling category and lactation number [41] (the number of cases entering the interval alive; the number of deaths in the interval; the proportion of deaths, i.e., the ratio of the number of deaths to the number of cases at risk in the interval; the cumulative proportion of surviving cows until the time interval (Sp) equal to the product of the survival probabilities of all preceding intervals; a probability density function (*f_i_*), i.e., the probability of culling in the time interval per unit time [39]:

(1) $$f_{i}=\frac{P_{i}-P_{i+1}}{h_{i}}$$

and the hazard rate (***γ****_i_*), i.e., the probability (per unit time) that a cow that survived to the start of the interval would be culled in this interval:

(2) $${\;\gamma }_{i}=\frac{f_{i}}{0.5\cdot \left(P_{i}+P_{i+1}\right)}$$

where *f_i_* is the probability density function, *P_i_* is the cumulative proportion of surviving cases at the beginning of the interval and the beginning of the subsequent interval (*P_i+_*_1_), *h_i_* is the time interval width).

Survival curves were compared for the first three lactations of cows culled for different reasons, recorded in the breeding documentation [41,49]. The Kaplan–Meier method was applied for the survival curve analysis. It estimates the survival function directly from the continuous survival time (the estimate is independent of data grouping) [14,33,49,50]. The probability estimate was calculated as the product of successive conditional probabilities estimated separately (with censored observations) [34,41].

The Kaplan–Meier estimator (Kaplan i. Meier, 1958) was estimated according to the following formula [34,37,39]:

(3) $$\hat{S}\left(t\right)=\prod_{t_{j}\leq t}\left(1-\frac{d_{j}}{r_{j}}\right)$$

where *r_j_* is the number of cows at risk at time *t_j_*, *d_j_* is the number of cows culled at time *t_j_*, Π is the product of all cases lower than or equal to *t*.

The significances between the survival curves were determined using the Cox–Mantel statistic (*C*), which (assuming the null hypothesis is true) has a standard normal distribution [43]:

(4) $$C=\frac{U}{\sqrt{I}}$$

(5) $$U=r_{2}-\overset{k}{\underset{i=1}{\sum }}m_{i}A\left(i\right)$$

(6) $$I=\overset{k}{\underset{i=1}{\sum }}\frac{m_{i}\left(r_{i}-m_{i}\right)}{r_{i}-1}\cdot A\left(i\right)\left(1-A\left(i\right)\right)$$

where *k* is the number of time moments in which cows were culled, *m_i_* is the number of cows culled at the *i*th time moment, *r_i_* is the number of observations from both groups being compared, in which the survival times are at least as long as or longer than the *i*th time moment, *A*(*i*) is the proportion of observations from Group 2, *r*_2_ is the total number of culled cows from Group 2.

Based on the Kaplan–Meier survival curves, survival probabilities (*P*1) were calculated for cows at the beginning of the age interval (*w*1), in which cow culling began, as well as at the end of the decrease when the curve stabilized for a given culling category (*P*2 and *w*2). The rate of decrease in survival probability was also calculated as the difference, i.e., *DP* = (*P*1 − *P*2) ∙ 100% and expressed as a percentage (*DP*).

For additional descriptions of cow survivability, regression model parameters were estimated and the values of the hazard ratio (*HR*) were determined over three successive lactations to identify the predictors most strongly correlated with the survival times of cows in each culling category [16,34,35,37,44,45,51].

The following variables were included in the model: calving season (1—autumn, 2—winter, 3—spring, 4—summer), the percentage of Holstein-Friesian genes (%), age at first calving (months), age at first breeding (months), the average number of days in milk during a complete lactation, the length of calving interval (days), the components of the production index used for the evaluation of Holstein-Friesian cows in Poland (production subindex (PSI), conformation subindex (CSI), fertility subindex (FSI), breeding value for somatic cell count (BVSCC), breeding value for longevity (BVL), body frame subindex (BFSI), strength and milk yield subindex (SMYSI), leg and hoof subindex (LHSI), udder subindex (USI), heifer conception rate (HCR), cow conception rate (CCR), the interval from calving to first insemination (CFI), calving-to-conception interval (CCI)), as well as milk performance traits (average daily milk yield (kg), the average milk fat content (%), and the average milk protein content (%)). The manner of calculating subindices is presented in Supplementary File S1. The partial maximum likelihood method was used for parameter estimation (an algorithm based on the Newton–Raphson iterative scheme) [52]. The Cox proportional hazards model was applied, which expresses the risk at time *t* for the analyzed set of independent variables (Cox, 1972):

(7) $$h\left(t:x_{1},x_{2},\ldots ,x_{k}\right)=h_{0}\left(t\right)e^{\overset{k}{\underset{i=1}{\sum }}a_{i}x_{i}}$$

where *h*(*t*:*x*_1_, *x*_2_,…*x_k_*) is the resulting hazard given *k* predictors and the appropriate survival time, *e* is the natural logarithm base, $\overset{k}{\underset{i=1}{\sum }}a_{i}x_{i}\;$ is the linear combination of independent variables (*x*_1_, *x*_2_…*x_k_*), and model parameters (*a*_1_, *a*_2_, …*a_k_*), *h*_0_(*t*) is the baseline hazard dependent on duration only.

The hazard ratio (*HR_i_*) was determined as:

(8) $$HR_{i}=e^{\overset{k}{\underset{i=1}{\sum }}a_{i}({\bar{x}}_{i}-x_{i})}$$

where $\overset{k}{\underset{i=1}{\sum }}a_{i}({\bar{x}}_{i}-x_{i})$ is the (summary) total effect of explanatory variables on the final risk (hazard) for a given cow, independent of duration, *a_i_* is the estimated regression coefficient.

*HR_i_* indicates the change in the risk of survival time shortening for one unit increase in the independent variable when adjusted for the remaining independent variables included in the model (it is assumed that they are constant when the independent variable increases by one unit). When *HR* > 1, the risk of survival time shortening increases, when *HR* < 1, the risk decreases. The values close to unity are interpreted as no risk. These values were calculated only for statistically significant regression coefficients.

In addition, a cluster analysis was carried out to show the similarities between cows culled for different reasons. The above-mentioned variables were used for the description of cull cows. The hierarchical Ward’s method was applied in the first stage of the cluster analysis [53]. It aims to obtain clusters with cases (culling categories) that are as similar as possible to each other and as different as possible from cases (culling categories) belonging to other clusters [54,55]. This can be obtained by merging all possible cluster pairs and selecting, each time, the cluster with the minimum sum of squared deviations [56,57,58,59] using an approach based on the analysis of variance to determine the distance between clusters [55,60,61,62,63]. The measure of the distance between cases (culling categories) and the mean value of a given cluster was the error sum of squares (*EES*), given by the following formula [64,65,66]:

(9) $$ESS=\overset{k}{\underset{i=1}{\sum }}{\left(x_{i}-\bar{x}\right)}^{2}$$

where *x_i_* is the value of the variable that is a clustering criterion for the *i*th case, *k* is the number of cases (culling categories) within the cluster, $\bar{x}$ is the mean value of this variable within the cluster.

The clusters created using Ward’s method were presented in the dendrogram showing the hierarchical structure of the set of culling categories [67] with decreasing similarities between them. Consequently, the analysis of the dendrogram revealed clusters formed by culling categories that were most similar to each other and represented by separate branches. The optimal number of clusters was finally determined through the pruning of the dendrogram. All the assumptions of Ward’s method were also verified [62,63].

To confirm the number of clusters determined with Ward’s method, the non-hierarchical *k*-means clustering algorithm was applied [47,48,68,69]. At first, all grouping variables were standardized [47,70] and the initial cluster centers were determined [68,71,72] by sorting all the distances between cases (culling categories), taking those at constant intervals [73]. Subsequently, the algorithm iteratively updated cluster centers [68,74] and the distances between these centers and individual cases (culling categories) to obtain the highest possible similarity between culling categories within clusters [71] and the highest possible degree of heterogeneity among clusters [60,69,75,76]. The Euclidean distance between culling categories and cluster centers was used [47,64,68,72]:

(10) $$d\left(x,y\right)=\sqrt{\overset{p}{\underset{i=1}{\sum }}{\left(x_{i}-y_{i}\right)}^{2}}$$

where ***x* =** (***x*_1_**, ***x*_2_**, …, ***x_p_***), ***y* =** (***y*_1_**, ***y*_2_**,…, ***y_p_***), *p* is the number of variables defining the *p*-dimensional space.

The number of clusters obtained with *k*-means clustering was the same as that determined with Ward’s method. All the assumptions of *k*-means clustering (i.e., the lack of collinearity among the grouping variables and the lack of outliers) were also verified [70,74,77,78,79].

All the calculations were carried out using Statistica 13.3 software (Tibco, Inc., Tulsa, OK, USA).

## 3. Results

### 3.1. Survival Tables and the Kaplan–Meier Survival Curves

Cows in their first lactation were most frequently culled between 24 and 31 months of age and the main culling reasons were udder diseases (about 20%) and locomotor system diseases (18%). The hazard rates ranged from 0.058 for udder diseases to 0.083 for metabolic and digestive system diseases (Supplementary Table S1). There were no cows culled due to contagious diseases. The first culling already occurred at about 20 months of age on average, whereas culling in older cows (above 31 months of age) was not observed in the first lactation.

**Figure 1:**
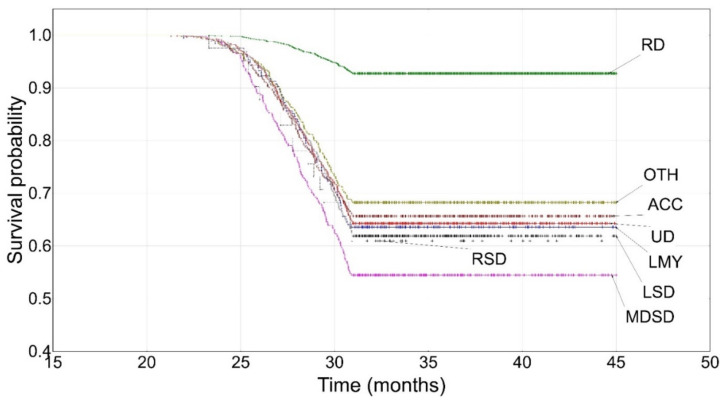
The Kaplan–Meier survival probability until the first lactation. UD—udder diseases, RD—reproductive disorders, MDSD—metabolic and digestive system diseases, RSD—respiratory system diseases, LSD—locomotor system diseases, ACC—accidents, OTH—others, LMY—low milk yield.

Based on the Kaplan–Meier functions, the survival curve decline could be observed at around 24–25 months of age (until about 31 months of age) for cows from individual culling categories (Figure 1). The smallest kink in the curve was noted only for cows culled due to reproductive problems (the difference in the cumulative proportions of surviving cases (*DP*) was about 7%). The sharpest kink could be seen for cows culled due to metabolic and digestive system diseases (*DP* = 43%) (Table 2). The shapes of these two curves were statistically significantly different from the curve shapes of cows from the remaining culling categories (Table 3). The survival probability for cows culled for different reasons ranged from 0.6094 for those from the respiratory system disease category to 0.9276 for those from the reproductive disorder category (Supplementary Table S2).

**Figure 2:**
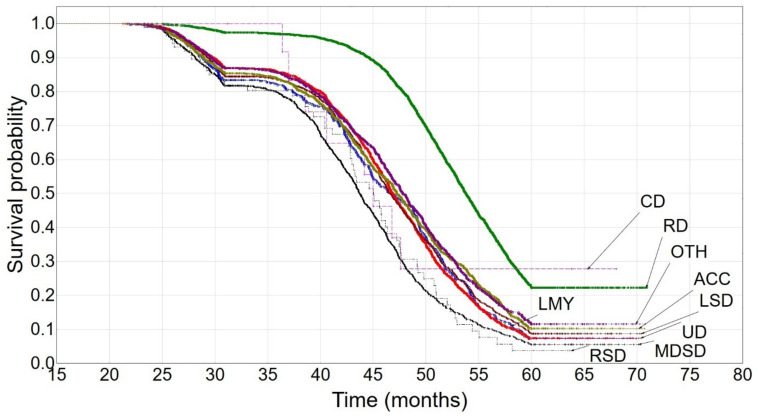
The Kaplan–Meier survival probability until the second lactation. UD—udder diseases, RD—reproductive disorders, CD—contagious diseases, MDSD—metabolic and digestive system diseases, RSD—respiratory system diseases, LSD—locomotor system diseases, ACC—accidents, OTH—others, LMY—low milk yield.

The highest culling number (until the second lactation, inclusive) was observed from 36 to 60 months of age and the most frequent culling categories were reproductive disorders (almost 33%) and udder diseases (17%). The hazard rates ranged from 0.08 to 0.12 (Supplementary Table S2). The lowest culling numbers were recorded for respiratory system diseases (0.8%) and contagious diseases (0.1%). The shallowest slope of the Kaplan–Meier survival curve was found for cows with reproductive problems (*DP* = 68%) (Figure 2). Only at around 45 months of age did a noticeable decline in the curve start, which stabilized at about 60 months of age at a survival probability of 0.2229. The survival curve for cows culled due to contagious diseases dropped sharply at around 38 months of age and stabilized ten months later at a survival probability of 0.2778. This curve was quite similar to that for cows culled due to respiratory system diseases; however, the latter stabilized later (at about 58 months of age) at a survival probability of 0.0394. The survival curves for cows culled due to low milk yield and udder diseases were also characterized by similar shapes. The decline was observed at about 36 months of age and stabilized at 60 months of age at a survival probability of 0.07. The curve for cows culled due to low milk yield additionally differed from that for cows culled for other reasons (the “others” category included those culling reasons that were difficult to classify to the remaining categories or more than one culling reason existed), whereas the curve for cows culled due to udder diseases was different from that for cows culled due to metabolic and digestive system diseases. The curves for cows culled for the remaining reasons declined at about 36 months of age and stabilized at around 60 months of age at a survival probability of 0.0553–0.1153.

**Figure 3:**
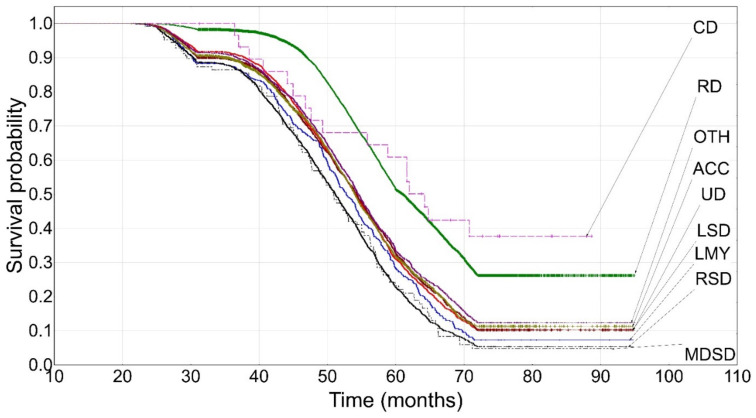
The Kaplan–Meier survival probability until the third lactation. UD—udder diseases, RD—reproductive disorders, CD—contagious diseases, MDSD—metabolic and digestive system diseases, RSD—respiratory system diseases, LSD—locomotor system diseases, ACC—accidents, OTH—others, LMY—low milk yield.

The highest culling number (until the third lactation, inclusive) was observed from 40 to 71 months of age. Cows culled due to reproductive disorders (32%) and udder diseases (almost 18%) predominated. The lowest culling number was recorded for contagious diseases (0.14%) and respiratory system diseases (0.74%). The hazard rates ranged from 0.041 to 0.073. The relatively shallow slope of the curve was characteristic of cows culled due to contagious diseases and reproductive disorders (Figure 3). The decline began at about 45 months of age and stabilized at around 70 months of age (*P*2 = 0.3771 for contagious diseases and *P*2 = 0.2620 for reproductive disorders; an approximately 63% decrease). The survival probability for the remaining curves ranged from 0.0394 (respiratory system diseases) to 0.1153 (others). The survival curve for cows with reproductive disorders (such as that for metabolic and digestive system diseases) was statistically significantly different from the remaining ones. It did not only differ from the curve for respiratory system diseases. The curves for cows with low milk yield were different from those for the “others” category and the curves for udder diseases differed from those for respiratory system diseases and accidents. The curve for locomotor system diseases was also different from the “others” category (Table 3).

The estimators obtained with the Kaplan–Meier method are independent of data grouping. Therefore, they do not directly correspond to the results from survival tables based on the Weibull distribution (Supplementary Tables S1–S3).

### 3.2. The Cox Proportional Hazards Model Parameters Affecting Cow Survival and Hazard Ratio (HR) Coefficients

The probability values indicating the statistical significance of the parameter estimates for the Cox proportional hazards model determined with the Wald statistic are shown in Table 4, together with the hazard ratio(s) (*HR*) showing the risk of survival time shortening according to different culling categories and lactation numbers. For cows culled due to low milk yield, statistically significant *HR* values until the first lactation ranged between 0.10 for the age at first calving and 1.26 for the percentage of HF genes. The respective values until the second and third lactations ranged from 0.53 to 1.03 and from 0.47 to 1.03 for the same two factors (temperament and BVL, respectively). For the udder disease category, statistically significant *HR* values until the first lactation ranged between 0.05 for the age at first calving and 1.02 for PSI. The respective values until the second and third lactations ranged from 0.44 for milk protein content to 1.23 for milk fat content and from 0.71 for temperament to 1.21 for milk fat content. For the reproductive disorder category, statistically significant *HR* values until the first lactation ranged between 0.30 for milk protein content and 1.03 for BVL. The respective values until the second and third lactations ranged from 0.34 for milk protein content to 1.36 for milk fat content and from 0.53 for milk protein content to 1.09 for CSI. No statistically significant parameters were found for the contagious disease category, except for milk fat content until the second lactation at a very high value of *HR* (19.41). For the metabolic and digestive system disease category, statistically significant *HR* values until the first lactation ranged between 0.09 for the age at first calving and 1.46 for temperament. The respective values until the second and third lactations ranged from 0.31 to 1.27 and from 0.26 to 1.30 for the same two factors (milk protein content and milk fat content, respectively). For the respiratory system disease category, statistically significant *HR* values until the first lactation ranged between 0.03 for the age at first calving and 0.88 for lactation length. The respective values until the second and third lactations ranged from 0.55 for the percentage of HF genes to 1.07 for BVL and from 0.86 for the age at first calving to 1.12 for BVL. For the locomotor system disease category, statistically significant *HR* values until the first lactation ranged between 0.22 for the age at first calving and 1.26 for USI. The respective values until the second and third lactations ranged from 0.33 for milk protein content to 1.08 for LHSI and from 0.59 for temperament to 1.20 for milk fat content. For the accident category, statistically significant *HR* values until the first lactation ranged between 0.32 for the age at first calving and 1.79 for FSI. The respective values until the second and third lactations ranged from 0.28 for milk protein content to 1.20 for FSI and from 0.26 for milk protein content to 1.20 for milk fat content. For the “others” category, statistically significant *HR* values until the first lactation ranged between 0.24 for the age at first calving and 1.11 for BFSI. The respective values until the second and third lactations ranged from 0.32 for milk protein content to 1.35 for milk fat content and from 0.37 for milk protein content to 1.22 for milk fat content and CSI.

### 3.3. Cluster Analysis

**Figure 4:**
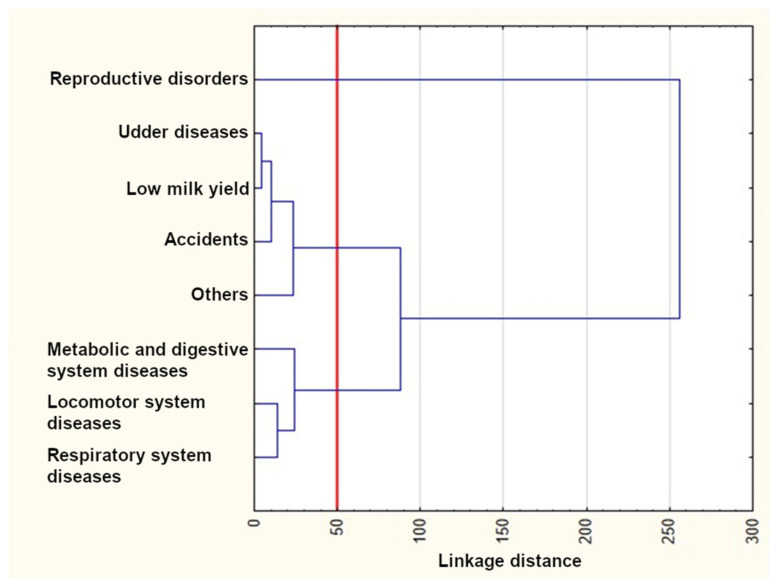
The results of the cluster analysis using Ward’s method for the first lactation.

**Figure 5:**
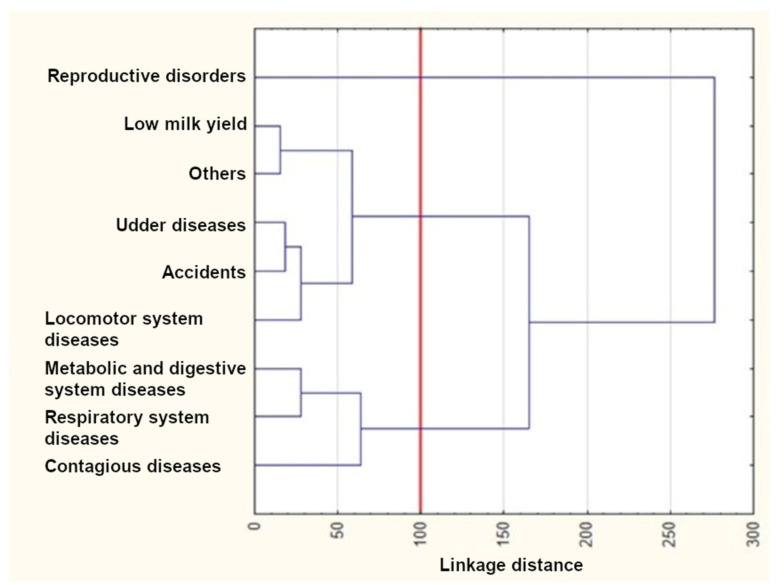
The results of the cluster analysis using Ward’s method for the first and second lactations.

**Figure 6:**
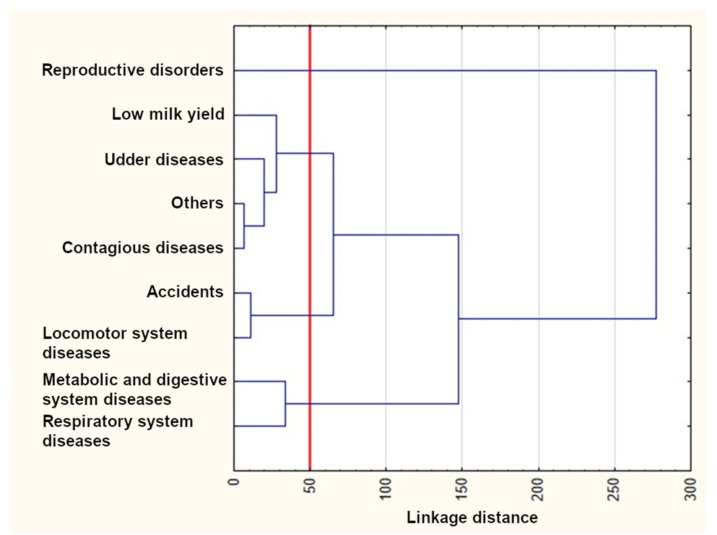
The results of the cluster analysis using Ward’s method for the first, second, and third lactations.

The results of the cluster analysis using Ward’s method are presented in Figure 4, Figure 5 and Figure 6. As can be seen from these figures, three clusters could be distinguished for the first lactation and the first two lactations, whereas four clusters were formed for the first three lactations. The reproductive disorder category clearly differed from the remaining ones and constituted a separate cluster in each of the three lactation groups. On the other hand, udder disease and low milk yield categories were similar and grouped in the same cluster in all dendrograms. Metabolic, digestive, and respiratory system diseases also occurred in one cluster for all lactation groups. The remaining categories rarely constituted separate clusters. In the third lactation group, locomotor system diseases and accidents formed a new separate cluster.

The *k*-means clustering confirmed the number of clusters determined with Ward’s method (Table 5), which means that the cluster members were exactly the same as those visible in the dendrogram. Three clusters existed until the first lactation. The first cluster included only reproductive disorders, whereas the second and third ones consisted of udder disease, low milk yield, accidents, and the “others” categories, as well as metabolic and digestive system diseases, locomotor system diseases, and respiratory system diseases, respectively. Three clusters could also be distinguished until the second lactation. The first one was the same as previously. The second cluster additionally included locomotor system diseases. The third cluster contained contagious diseases instead of locomotor system diseases. Four clusters existed until the third lactation. The first one was identical as previously. The second cluster was similar to those of the first two lactation groups; however, it included contagious diseases instead of accidents (such as that for the first lactation group) or accidents and locomotor system diseases (such as that for the second lactation group). Finally, the last two clusters consisted of accidents and locomotor system diseases as well as metabolic and digestive system diseases and respiratory system diseases, respectively.

## 4. Discussion

As already mentioned, an important feature of survival analysis is the fact that information from cows that are not culled by the time the data recording is complete can also be used. Cows still alive at the end of the study period may cause difficulties in survival evaluation using other statistical methods since the actual life spans of the animals are not known. Therefore, the survival analysis offers numerous advantages over traditional linear models and allows for better fitting the survival data and using time-dependent variables appropriately [14,19,42]. The cluster analysis, on the other hand, creates homogenous groups of culling reasons. Cows have increased chances of remaining in the herd if they are healthy, reproduce regularly, have functional feet, legs, and udders, and produce enough milk [15]. Thus, knowledge of the association between various indicators monitored at the individual animal and herd levels with cow lifespans and culling reasons is essential to predict cow longevity and support optimal decisions in herd management [31]. The survival and cluster analyses performed in the present study showed some similarities between individual culling categories, which may indicate their direct connection. For instance, the survival curve for contagious diseases was similar to that for respiratory system diseases in the second lactation group (both categories grouped in the same cluster), which may suggest that pathogens causing respiratory system infections can finally lead to cow culling. The survival curve for low milk yield was also similar to that for udder diseases in the same lactation group (both included in the same cluster), which may imply that the milk yield of cows with udder diseases (such as mastitis) decreases, ultimately resulting in their culling. Finally, the reproductive disorder category clearly differed from the remaining ones and formed a separate cluster. A more detailed description of the similarities and differences between individual survival curves and clusters and their consequences is given below.

The main culling categories analyzed in the present study (reproductive disorders—40%, udder diseases—13 to 15%, and locomotor system diseases—above 10%) corresponded to the frequency of culling reasons reported by others [15,24,80,81,82,83,84], although Reimus et al. [85] identified leg problems as the main culling category (above 26%). Similarly, Sewalem et al. [86] and Zavadilová et al. [87] indicated udder and leg diseases and all the resulting consequences as the most important culling reasons. The effect of lameness on culling is not unequivocal. Milian-Suazo et al. [88] and Barkema et al. [89] argued that lameness affected culling to a small extent. According to Gröhn et al. [90], the decision on culling due to lameness was mainly associated with calving age, milk yield, and fertility. Therefore, numerous variables should be included in the analysis of the influence of lameness on culling risk. Kugonza et al. [91] indicated fertility, poor productivity, health performance, as well as old age as the main factors affecting the culling rate. The remaining reasons (identified with the questionnaires on the long-horned Ankole cattle) included inbreeding, production of males only, bad temperament, high calf mortality, slow growth, unfavorable color, inappropriate conformation, and body condition. Ahlman et al. [92] analyzed six longevity traits in Swedish Holstein and Swedish Red cows (the length of productive life; survival through the first, second, and third lactations; fertility- and udder health-determined survival) and divided culling reasons into eight groups: udder health, low fertility, low production, leg problems, metabolic diseases, other diseases, other specified causes, and unspecified causes. Finally, economic factors affecting the culling rate include input and production prices and their seasonal variations [93,94].

The survival curves for cows from the reproductive disorder category clearly differed from the remaining ones in each of the three lactation groups (Figure 1, Figure 2 and Figure 3 and Table 3) and constituted a separate cluster (Figure 4, Figure 5 and Figure 6). Moreover, the curves for udder diseases and low milk yield were very similar (they differed significantly only for the third lactation) and were grouped in the same cluster. Furthermore, the survival curves for cows from the metabolic and digestive system disease category were different from most survival curves for the remaining culling categories (except for respiratory system diseases). The cluster analysis showed that digestive and respiratory system diseases occurred in one cluster for all lactations. In addition, the reproductive disorder category constituted a separate cluster for each of the three lactations. It should be emphasized that reproductive factors have low heritability, which means that their contribution to the variability of this trait is not great. It may suggest that not all cows culled due to reproductive problems were infertile [95]. The cows from the remaining categories rarely constituted separate clusters, which was also confirmed by the analysis of the differences among survival curves (Table 3). In the first lactation, the low milk yield and udder disease clusters could also be distinguished. It can be noticed that the three main clusters of cull cows were formed until the second lactation, whereas four clusters existed until the third lactation. It is also noteworthy that locomotor system diseases and accidents constituted a new separate cluster. This connection may suggest that the locomotor system disease and accident categories may have a lot in common; the health status of cows that are culled due to locomotor system diseases and accidents should be verified more precisely since accidents can implicate leg diseases or vice versa. Booth et al. [96] indicated that the culling risk due to lameness may be underestimated unless the temporal relationship between lameness and culling is included in the model. It results from the fact that such a model assumes that the culling risk of a lame cow is constant in time, even before the occurrence of lameness. On the other hand, Ahlman et al. [92] reported that low fertility was the main culling reason in the first lactation, regardless of breed or production system, whereas the change in the main culling cause from fertility (most common in the first lactation, irrespective of the production system) to udder health occurred at a lower age in organic herds.

Table 4 presents the relative hazard ratio(s) (*HR*) of culling a cow from individual culling categories, taking into account different factors. Fodor et al. [97] reported that the high age at first calving in heifers (above 30 months) increased culling probability by more than five times compared to that of less than 22 months. In the present study, the *HR* for the age at first calving was below unity practically for each culling category, which would suggest an increased survival chance with a one-month increase in the age at first calving. This result corresponds to the values close to 0.99 for a one-day increase in the age at first calving reported by Whitaker et al. [98]. However, it should be noticed that the age at first calving is characterized by a certain optimum range (22 to 25 months) and the values below or above this threshold contribute to a higher culling probability [99].

Calving season was practically insignificant for an increased culling risk in each culling category, except for udder diseases in the second lactation, in which this risk was slightly higher in the spring–summer season (*HR* > 1) and the “others” category, where it decreased in the same season (*HR* < 1). Bradley and Green [100] suggested that culling risk due to udder diseases increased over the autumn–winter period. Some authors indicated that the summer calving season reduced cow survival [95,98].

Gröhn et al. [90] reported that a higher milk yield decreased the culling rate due to udder diseases, reproductive problems, and metabolic diseases, which corresponds to the results of the present study to some extent. The *HR* values for milk yield were close to unity or slightly lower for these culling reasons. This coefficient was similar to that for SMYSI in the above-mentioned culling categories.

A significant association between locomotor system disease category and many variables was found. Increased age at first calving decreased the culling risk in each of the three lactations. Moreover, increased lactation length, temperament (in the second and third lactations), and the percentage of milk protein had the same effect. On the other hand, BFSI caused a higher culling risk in the first and second lactations (*HR* = 1.11 and *HR* = 1.05, respectively). Similarly, CSI, as a whole, increased this risk, especially in heifers (*HR* = 1.55). The same situation was observed for milk fat content in the third lactation (*HR* = 1.2) and USI (including traits such as: udder placement, fore udder attachment, rear udder attachment, medial suspensory ligament, udder width, rear teat placement, fore teat placement, and teat length) in the first lactation (*HR* = 1.26). Other traits had low *HR* values or their effects on culling risks were statistically non-significant.

The influence of exterior traits on the length of productive life in Slovak Simmental cows was analyzed by Čanji et al. [101] and Strapák et al. [102]. The udder had a great impact on this length among the main exterior traits. From the partial exterior traits, the largest effect on the length of productive life was observed for udder depth, teat length, thickness, and placement. Similar results were obtained by Strapák et al. [103], who found that udder depth, teat length, rear udder attachment, and the rear udder significantly influenced the length of productive life. In the study by Strapáková et al. [104] on Slovak Simmental cows, whose body conformation traits were evaluated according to the Fleckscore system, animals with deeper udders, stronger and tighter fore udder attachments, and centrally placed teats, achieved longer productive lives. The effect of udder traits on the culling rate was also observed in Slovak Spotted cattle by Strapák et al. [105]. The relationship between conformation traits and longevity indicators in Polish Holstein-Friesian Black-and-White cows was analyzed by Sawa et al. [106], who found that lifespan, the length of productive life, and the number of lactations depended primarily on udder conformation (udder as a general trait and conformation score) (*r* = 0.11) and, among detailed traits, on udder placement (*r* = 0.14) and fore udder attachment (*r* = 0.10). Vacek et al. [107], investigating the associations between conformation traits and longevity in Czech Holstein cattle, reported that rump angle, rear leg set, udder depth, and teat length were negatively correlated with the herd or productive life. Most body traits were weakly (but positively) associated with herd life, indicating that larger cows lived longer. However, body depth and chest width were not linearly related to longevity. The longest productive lives were observed in cows undersized in chest width and body depth and a similar dependence was found for rump width. The ideal rear leg set for longevity was average or below average. Cows with well-attached fore udders, high-attached rear udders, strong central ligaments, close front teat placement, and moderately long teats showed the longest functional productive lives.

Studies by Čanji et al. [101], Strapák et al. [102], and Strapák et al. [103] also highlighted the effect of body frame on the culling rate in Slovak Simmental cattle. Among body conformation traits, rump angle, croup height, and body depth were the main factors affecting this rate. Strapáková et al. [104] found that smaller, longer, and well-muscled cows with deeper bodies had a lower risk of early culling, whereas hock angularity and pastern and hoof height with low or high scores increased this risk. Finally, the influence of body conformation traits on longevity in Slovak Spotted cows was investigated by Strapák et al. [105], who reported that body frame significantly affected the length of productive life in this breed.

According to Booth et al. [96], foot rot diagnosed during the second or third months of lactation decreased survival during the same time period (hazard ratio = 5.1; 95% confidence interval = 1.6 to 16.2). Sole ulcers occurring in the first four months of lactation reduced survival in several subsequent periods, in which the strongest association was observed between diagnosis in the third and fourth months of lactation and exit from the herd during the same period (hazard ratio = 2.7; 95% confidence interval = 1.3 to 6.0). In the study by Strapák et al. [102], feet and legs belonged to the main exterior traits affecting the culling rate. A more detailed analysis of the partial exterior traits showed that heel joint expression and feet significantly influenced the culling risk in Slovak Simmental cows. Similar results were obtained by Čanji et al. [101] and Strapák et al. [103], who found that fetlock, feet, and rear legs were the most important traits affecting the length of productive life. In the study by Sawa et al. [106], the conformation of legs and feet correlated with lifespan, the length of productive life, and the number of lactations (*r* = 0.11). Cramer et al. [108] included heifers, infectious lesions, white line lesions, hemorrhages, sole ulcers, other lesions, and free-stall housing as covariates in the final multivariate Cox proportional hazards model. Infectious hoof lesions were not significantly associated with culling probability. Hazard ratios for white line lesions, ulcers, and hemorrhages were 1.72, 1.26, and 1.36, respectively. The strength of the association between the grouped variables (other lesions) and culling risk decreased with time.

In the present study, the *HR* value for the percentage of milk protein was below unity for almost all culling categories, which suggested a decreased culling risk. A similar effect of increased milk protein content on culling rate was observed by Morek-Kopeć et al. [109], indicating that a lower protein content associated with the lactation stage and difficult calving may contribute to the culling of a cow. An increased percentage of milk fat was related to the higher culling risk for most culling categories, especially in the second and third lactations. A similar advantageous effect of protein content and a more neutral influence of fat content on the culling rate was found by Sasaki et al. [110], Chirinos et al. [111], and Morek-Kopeć et al. [109].

## 5. Conclusions

The identification of early predictors of a cow’s productive life is of great significance to improve dairy herd profitability. The supply of quality replacement heifers, mitigation of transition cow disease risk, and good culling decisions have great impacts on the productivity and profitability of the herd. Culling decisions are made based on cows and external factors that are frequently specific to the herd. The survival analysis provides information about the potential of cows characterized by certain traits for breeders. It supports more rational management of reproduction and performance and the application of preventive measures that allow for expanding production and eliminating disadvantageous phenomena. The survival analysis may also provide some information about the size of the future population, enabling a better understanding of survival patterns according to population age, and determining the effects of different factors on these patterns, which was presented and confirmed in the present study. The survival curves for cows from individual culling categories had similar shapes, although those for the reproductive disorder category (especially in the first lactation) and the contagious disease category (in the second and third lactations) had shallower slopes. The low level of probability of survival curves for cows from the metabolic and digestive system disease category and the respiratory system disease category could also be observed in each of the three lactations. The contagious disease category was almost non-existent in the first lactation.

The culling reason was not always precisely determined for the distinguished culling categories, which was confirmed by some results, e.g., the accident and locomotor system disease categories. A more accurate method of determining culling reasons would be required, especially for these two categories.

Among the indices from the breeding documentation included in the analysis, the greatest effect on the relative culling risk in individual lactations was observed for age at first calving, lactation length, calving interval, PSI, BVL, temperament, and average daily milk yield, whereas the smallest one was found for calving season, BFSI, SMYSI, CCR, CFI, and CCI.

## Figures and Tables

**Table 1:** Culling categories between 2017 and 2018 according to the breeding documentation in the original dataset and the selected subset.

	Lactation 1				Lactation 2				Lactation 3			
Class	Original Dataset		Subset		Original Dataset		Subset		Original Dataset		Subset	
	*n*	%	*n*	%	*n*	%	*n*	%	*n*	%	*n*	%
LMY ^1^	2849	3.72	195	3.97	5416	3.38	427	3.40	7323	3.15	608	3.12
UD ^2^	9973	13.03	686	13.98	22,811	14.23	1862	14.80	35,205	15.12	2990	15.36
RD ^3^	33,619	43.92	1878	38.28	69,526	43.36	5090	40.47	97,116	41.71	7741	39.76
CD ^4^	121	0.16	2	0.04	251	0.16	13	0.10	352	0.15	30	0.15
MDSD ^5^	5654	7.39	435	8.87	12,621	7.87	1085	8.63	19,598	8.42	1746	8.97
RSD ^6^	533	0.70	41	0.84	1060	0.66	87	0.69	1485	0.64	126	0.65
LSD ^7^	8062	10.53	591	12.05	16,807	10.48	1438	11.43	25,347	10.89	2250	11.56
ACC ^8^	8177	10.68	577	11.76	16,455	10.26	1360	10.81	24,109	10.36	2109	10.83
OTH ^9^	7552	9.87	501	10.21	15,383	9.59	1215	9.66	22,274	9.57	1870	9.60

^1^ low milk yield, ^2^ udder diseases, ^3^ reproductive disorders, ^4^ contagious diseases, ^5^ metabolic and digestive system diseases, ^6^ respiratory system diseases, ^7^ locomotor system diseases, ^8^ accidents, ^9^ others (the “others” category included those culling reasons that were difficult to classify to the remaining categories or more than one culling reason existed).

**Table 2:** Cumulative proportions of cows surviving (*P1* and *P2*) until the age interval (*w1* and *w2*) and the percentage decrease (*DP*).

Reason	Until the First Lactation					Until the Second Lactation					Until the Third Lactation				
	*P*1	*w*1	*P*2	*w*2	*DP*	*P*1	*w*1	*P*2	*w*2	*DP*	*P*1	*w*1	*P*2	*w*2	*DP*
LMY ^1^	0.9708	24	0.6364	31	33.44	0.8000	36	0.0742	60	72.58	0.8325	40	0.0742	71	75.83
UD ^2^	0.9718	24	0.6429	31	32.89	0.8280	36	0.0742	60	75.38	0.8613	40	0.0742	71	78.71
RD ^3^	0.9973	25	0.9276	31	6.97	0.9001	40	0.2229	60	67.72	0.9012	40	0.2620	70	63.92
CD ^4^	-	-	-	-	-	0.9999	38	0.2778	47	72.21	0.9999	40	0.3771	70	62.28
MDSD ^5^	0.9711	24	0.5455	31	42.56	0.8000	36	0.0553	60	74.47	0.8207	40	0.0553	71	76.54
RSD ^6^	0.9705	24	0.6098	31	36.07	0.7772	36	0.0394	58	73.78	0.8206	40	0.0394	71	78.12
LSD ^7^	0.9722	24	0.6201	31	35.21	0.8280	36	0.0872	60	74.08	0.8613	40	0.0872	71	77.41
ACC ^8^	0.9721	24	0.6521	31	32.00	0.8532	36	0.1020	60	75.12	0.8613	40	0.1020	71	75.93
OTH ^9^	0.9705	24	0.6751	31	29.54	0.8557	36	0.1153	60	74.04	0.8676	40	0.1153	71	75.23

^1^ low milk yield, ^2^ udder diseases, ^3^ reproductive disorders, ^4^ contagious diseases, ^5^ metabolic and digestive system diseases, ^6^ respiratory system diseases, ^7^ locomotor system diseases, ^8^ accidents, ^9^ others (the “others” category included those culling reasons that were difficult to classify to the remaining categories or more than one culling reason existed), *P*1—the cumulative proportion of cows surviving until the interval in which culling started; *P*2—the cumulative proportion of cows surviving until the interval in which culling ended; *w*1—the month of life of a cow, in which culling started for a given category; *w*2—the month of life of a cow, in which culling ended for a given lactation; *DP*—the rate of decrease = (*P*1 − *P*2) · 100%; the values determined from Kaplan–Meier curves.

**Table 3:** *p*-values for comparisons between survival curves for cows from individual culling categories.

First Lactation								
Category	UD ^1^	RD ^2^	CD ^3^	MDSD ^4^	RSD ^5^	LSD ^6^	ACC ^7^	OTH ^8^
LMY ^9^	0.8505	0.0000		0.0265	0.7102	0.7330	0.7049	0.2597
UD		0.0000		0.0005	0.6206	0.4351	0.7622	0.1799
RD				0.0000	0.0000	0.0000	0.0000	0.0000
MDSD					0.4333	0.0067	0.0003	0.0000
RSD						0.8199	0.5470	0.3106
LSD							0.3071	0.0440
ACC								0.3150
Until the Second Lactation								
LMY	0.7113	0.0000	0.2995	0.0001	0.0789	0.5368	0.1325	0.0236
UD		0.0000	0.3344	0.0000	0.0378	0.6205	0.0534	0.1591
RD			0.0037	0.0000	0.0000	0.0000	0.0000	0.0000
CD				0.1073	0.1750	0.3661	0.4543	0.5868
MDSD					0.8175	0.0000	0.0000	0.0000
RSD						0.0936	0.0762	0.0171
LSD							0.1800	0.0356
ACC								0.2776
Until the Third Lactation								
LMY	0.0110	0.0000	0.0026	0.0247	0.2958	0.0329	0.0402	0.0022
UD		0.0000	0.0098	0.0000	0.0136	0.3608	0.5817	0.2602
RD			0.8454	0.0000	0.0000	0.0000	0.0000	0.0000
CD				0.0003	0.0015	0.0087	0.0107	0.0205
MDSD					0.9918	0.0000	0.0000	0.0000
RSD						0.0237	0.0273	0.0062
LSD							0.9185	0.1517
ACC								0.1325

^1^ udder diseases, ^2^ reproductive disorders, ^3^ contagious diseases, ^4^ metabolic and digestive system diseases, ^5^ respiratory system diseases, ^6^ locomotor system diseases, ^7^ accidents, ^8^ others, ^9^ low milk yield.

**Table 4:** *HR* values for the individual parameters of the Cox proportional hazards model according to culling categories and lactation groups (1—until the first lactation, 2—until the second lactation, 3—until the third lactation; *HR* values for statistically significant parameters are marked in bold).

Category	LMY ^1^			UD ^2^			RD ^3^			CD ^4^		MDSD ^5^			RSD ^6^			LSD ^7^			ACC ^8^			OTH ^9^		
Lactation	1	2	3	1	2	3	1	2	3	2	3	1	2	3	1	2	3	1	2	3	1	2	3	1	2	3
**CS ^10^**	1.04	1.07	0.98	0.95	**1.08**	0.98	1.15	0.99	0.99	1.52	1.22	0.89	1.03	1.00	0.45	0.84	1.04	0.99	1.01	1.04	0.87	0.95	1.00	**0.73**	1.04	1.00
**HF ^11^**	**1.26**	1.00	0.99	1.00	0.99	**0.98**	0.95	0.99	0.98			0.98	0.99	1.00	0.16	**0.55**	0.77	1.03	0.99	0.99	**0.94**	**0.97**	**0.97**	0.98	0.97	0.99
**AFC ^12^**	**0.10**	**0.82**	0.89	**0.05**	**0.78**	**0.85**	**0.40**	**0.82**	**0.86**	0.87	0.96	**0.09**	**0.77**	**0.88**	**0.03**	**0.59**	**0.86**	**0.22**	**0.77**	**0.85**	**0.32**	**0.77**	**0.84**	**0.24**	**0.80**	**0.86**
**AFB ^13^**	0.87	0.99	0.98	1.01	**1.03**	**1.02**	0.97	1.00	0.99	1.78	0.98	0.99	0.99	0.99	0.98	0.95	0.94	**0.92**	1.03	**1.03**	0.98	**1.04**	1.02	0.87	1.01	1.02
**LL ^14^**	**0.92**	**0.99**	**1.00**	**0.91**	**0.99**	**1.00**	**0.96**	1.00	**1.00**	0.96	1.00	**0.90**	**0.99**	**1.00**	**0.88**	**0.99**	**1.00**	**0.94**	**0.99**	**1.00**	**0.96**	**0.99**	**1.00**	**0.94**	**0.99**	**1.00**
**CI ^15^**		**0.99**	**1.00**		**0.99**	**1.00**		**0.99**	**0.99**	1.01	0.99		**0.99**	**1.00**		**0.99**	**0.99**		**0.99**	**1.00**		**1.00**	**1.00**		**0.99**	**1.00**
**PSI ^16^**	0.98	1.02	**1.02**	**1.02**	**0.99**	**1.02**	1.01	**0.98**	1.00	0.98	1.16	1.00	**1.02**	**1.02**	1.17	0.99	1.00	1.02	1.01	**1.01**	1.00	**1.02**	**1.02**	0.97	1.00	**1.02**
**CSI ^17^**	0.96	1.01	1.01	0.87	1.11	1.03	0.67	0.97	**1.09**	0.85	0.01	1.22	1.05	**1.13**	1.02	1.11	0.71	0.71	0.87	1.13	**1.59**	0.88	1.05	0.95	1.19	**1.22**
**BFSI ^18^**	0.94	1.00	1.00	1.05	0.99	1.01	1.01	1.01	0.99	1.05	2.63	0.98	1.00	0.99	0.98	0.95	1.06	**1.11**	**1.05**	0.99	0.97	1.02	1.00	**1.11**	0.98	0.98
**SMYSI ^19^**	1.10	1.00	1.00	1.02	0.99	1.00	1.08	1.01	1.00	0.88	2.07	0.97	1.01	1.00	1.06	1.01	1.11	1.05	1.01	1.01	0.95	1.03	1.02	**0.91**	0.98	0.99
**LHSI ^20^**	0.97	0.98	0.99	1.09	0.96	0.99	1.17	1.01	**0.97**	1.55	11.30	0.94	**0.97**	**0.95**	1.05	1.00	1.14	1.13	**1.08**	0.96	**0.85**	1.06	0.98	1.07	0.95	**0.93**
**USI ^21^**	0.91	1.00	1.00	1.05	0.92	0.98	1.37	1.03	**0.96**	0.88	25.26	0.87	0.98	**0.92**	1.04	0.91	1.30	**1.26**	1.10	0.92	**0.73**	1.11	0.98	1.09	0.91	**0.88**
**FSI ^22^**	1.08	1.25	0.97	1.08	0.99	1.01	1.04	0.99	1.01	0.11	0.14	0.85	0.96	0.98	1.02	1.13	1.18	1.13	1.01	**1.02**	**1.79**	**1.20**	**1.09**	0.95	1.13	1.11
**HCR ^23^**	0.91	0.81	1.03	0.93	1.00	**0.98**	1.04	1.00	0.99	7.68	6.13	1.19	1.03	1.01	1.06	0.89	0.85	0.89	**0.97**	**0.97**	**0.62**	**0.83**	**0.91**	1.03	**0.86**	**0.89**
**CCR ^24^**	0.95	0.98	0.99	0.97	1.00	1.00	0.95	1.00	0.99	1.14	1.21	0.98	1.00	**1.02**	0.98	0.97	1.02	0.97	**1.02**	1.01	**0.89**	0.98	1.00	1.03	1.01	0.99
**CFI ^25^**	0.96	0.99	0.96	0.98	**0.97**	0.98	1.01	1.01	1.00	1.18	1.93	1.05	1.00	1.02	1.05	0.98	1.03	0.97	1.00	1.01	**0.86**	0.98	1.01	1.02	0.97	0.98
**CCI ^26^**		0.95	1.06		1.02	1.02		0.99	**1.01**	1.10	0.96		1.02	0.99		1.03	0.91		1.00	0.99		0.98	0.98		1.01	1.00
**BVSCC ^27^**	0.95	1.01	1.01	1.00	0.99	**1.01**	1.00	1.00	**0.99**	0.82	1.21	1.00	**1.01**	1.00	0.99	0.96	1.00	**1.05**	**1.01**	1.00	**0.95**	**0.98**	**0.99**	**0.97**	1.00	1.00
**BVL ^28^**	**1.08**	**1.03**	**1.03**	1.00	**1.03**	**1.03**	**1.03**	**1.02**	**1.02**	1.11	0.87	**1.02**	**1.02**	**1.03**	1.05	**1.07**	**1.12**	1.00	1.01	**1.03**	**1.05**	**1.03**	**1.03**	**0.97**	1.01	**1.02**
**Temp ^29^**	0.83	**0.53**	**0.47**	**0.79**	**0.63**	**0.71**	0.79	**0.81**	0.94	2.72	0.42	**1.46**	**0.47**	**0.49**	**0.38**	0.88	0.62	1.13	**0.51**	**0.59**	**0.81**	**0.61**	**0.58**	**0.81**	**0.70**	**0.67**
**MY ^30^**	0.98	**0.96**	**0.96**	**0.96**	1.01	**0.98**	1.02	**1.02**	**0.99**	1.17	0.77	1.02	**0.97**	**0.97**	1.05	1.00	0.97	1.00	0.99	**0.98**	1.00	**0.98**	**0.98**	**1.04**	1.00	0.99
**Fat ^31^**	0.79	1.12	0.92	1.00	**1.23**	**1.21**	0.89	**1.36**	1.05	**19.41**	2.10	1.01	**1.27**	**1.30**	2.05	1.64	1.17	0.90	1.12	**1.20**	1.08	**1.18**	**1.20**	0.84	**1.35**	**1.22**
**Prot ^32^**	0.90	0.72	0.76	0.90	**0.44**	0.17	**0.30**	**0.34**	**0.53**	0.00	0.00	0.93	**0.31**	**0.26**	0.22	0.37	0.46	**0.66**	**0.33**	0.36	0.85	**0.28**	**0.26**	1.08	**0.32**	**0.37**

^1^ low milk yield, ^2^ udder diseases, ^3^ reproductive disorders, ^4^ contagious diseases, ^5^ metabolic and digestive system diseases, ^6^ respiratory system diseases, ^7^ locomotor system diseases, ^8^ accidents, ^9^ others, ^10^ calving season, ^11^ percentage of HF genes, ^12^ age at first calving, ^13^ age at first breeding, ^14^ lactation length, ^15^ calving interval, ^16^ production subindex, ^17^ conformation subindex, ^18^ body frame subindex, ^19^ strength and milk yield subindex, ^20^ leg and hoof subindex, ^21^ udder subindex, ^22^ fertility subindex, ^23^ heifer conception rate, ^24^ cow conception rate, ^25^ the interval from calving to first insemination, ^26^ calving-to-conception interval, ^27^ breeding value for somatic cell count, ^28^ breeding value for longevity, ^29^ temperament, ^30^ milk yield, ^31^ fat percentage, ^32^ protein percentage.

**Table 5:** Cluster elements for the *k*-means clustering method.

Cluster Number	Culling Category
First lactation	
1	Reproductive disorders
2	Udder diseases
	Others
	Low milk yield
	Accidents
3	Metabolic and digestive system diseases
	Locomotor system diseases
	Respiratory system diseases
First and second lactation	
1	Reproductive disorders
2	Udder diseases
	Others
	Low milk yield
	Accidents
	Locomotor system diseases
3	Metabolic and digestive system diseases
	Respiratory system diseases
	Contagious diseases
First, second and third lactation	
1	Reproductive disorders
2	Udder diseases
	Others
	Low milk yield
	Contagious diseases
3	Accidents
	Locomotor system diseases
4	Metabolic and digestive system diseases
	Respiratory system diseases

## Data Availability

The data are available upon reasonable request from the corresponding author. The data set was provided by the Polish Federation of Cattle Breeders and Dairy Farmers in Warsaw (PFCBDF); Contract No. 47/2013, 36/2016 and PN/147/2019 between PFCBDF and the University of Agriculture in Krakow; the purchase of the data was financed from the funds for statutory activity, by the Ministry of Science and Higher Education in Poland.

## Supplementary Materials

The following supporting information can be downloaded at: https://www.mdpi.com/article/10.3390/ani12151942/s1, Table S1: Survival tables for cows until the first lactation according to culling categories; Table S2: Survival tables for cows until the second lactation according to culling categories; Table S3: Survival tables for cows until the third lactation according to culling categories; Supplementary File S1: The PF (Productivity and Functionality) selection index.
