# Supplementary material for: Estimation of Dairy Cow Survival in the First Three Lactations for Different Culling Reasons Using the Kaplan–Meier Method
Source: Animals (Basel). 2022 Jul 30;12(15):1942. doi: 10.3390/ani12151942 (PMC9367421; doi:10.3390/ani12151942)
Supplement: Supplementary file 1 [file animals-12-01942-s001.zip › animals-1824023-supplementary.pdf]

Supplementary File S1

PF—Polish Holstein–Friesian breed.

The productivity and functionality (PF) selection index, effective since 2007 for Polish Holstein–Friesian bulls, was modified in 2014 and adjusted to the current needs and breeding aims realized by breeders. The PF index is given by the following formula:

PF = 0.4* PSI + 0.25* CSI + 0.15* FSI + 0.1* BVSCC+ 0.1* BVL

where: PSI—production subindex, CSI—conformation subindex, FSI—fertility subindex, BVSCC—breeding value for somatic cell count, BVL—breeding value for longevity.

PSI—consists of the sum of the breeding value for the fat yield (kg) and the doubled breeding value for the protein yield (kg). Before the creation of this subindex, the values included in it were standardized with a mean of 100 and a standard deviation of 10, taking the mean breeding value for bulls born between 2004 and 2006 with at least 20 daughters from 10 herds as a basis.

CSI—was created from the following partial subindices with the following weights for each of them:

- USI—udder subindex—50%.
- LHSI—leg and hoof subindex—30%.
- SMYSI—strength and milk yield subindex—10%.
- BFSI—body frame subindex—10%.

Udder subindex:

- Udder placement—35%.
- Fore udder attachment—18%.
- Rear udder attachment—15%.
- Medial suspensory ligament—10%.
- Udder width—10%.
- Rear teat placement—6%.
- Fore teat placement—3%.
- Teat length—3%.

Leg and hoof subindex:

- Hoof diagonal—45%.
- Leg set (rear view)—35%.
- Leg set (lateral view)—20%.

Strength and milk yield subindex:

- Dairy character—50%.
- Chest width—25%.
- Body depth—15%.
- Hip height—10%.

Body frame subindex:

- Rump angle—40%.
- Hip height—25%.
- Rump width—20%.
- Chest width—15%.

FSI—four estimated breeding values for fertility traits affect this subindex with the following weights:

- Heifer conception rate (HCR)—70%.
- Cow conception rate (CCR)—10%.
- The interval from calving to first insemination (CFI)—10%.
- Calving-to-conception interval (CCI)—10%.

Heifer or cow conception rate (CR) is calculated based on all insemination services, i.e., from the first to the effective one, according to

the following formula: 100/number of services. The more times the female was inseminated, the lower the conception rate.

- CR = 100 for the first effective insemination.
- CR = 50 for the second effective insemination.
- CR = 25 for the fourth effective insemination.

It is very important that all insemination services are recorded, from the first to the effective one.

BVSCC—is estimated with the TDM method based on the somatic cell count in the milk from individual test days during the first three lactations. The bull breeding values higher than 100 indicate that the bull improves this trait in his daughters.

BVL—longevity is defined as the difference expressed in days between the culling date for culled cows or the last recorded test day for cows still alive and the first calving date. The mean breeding value for bulls born between 2004 and 2006 with the evaluation repeatability of at least 50% is taken as the basis. The condition for the confirmation and publication of the breeding values for longevity is the evaluation repeatability of at least 20%.

- BVL = 100 – 0.5* (BBV - 100) + 0.25* (GBV - 100).
- BBV—bull breeding value.
- GBV—maternal grandsire breeding value.

Table S1. Survival tables for cows until the first lactation according to the culling categories.

X_1	H_i	l_1	L_Z	PZ	Sp	f_i	h_i	X_1	H_i	l_1	L_Z	PZ	Sp	f_i	h_i
**LOW MILK YIELD**								**RESPIRATORY SYSTEM DISEASES**							
0.0	5.0	195	0	0.0026	1.0000	0.0005	0.0005	0.0	4.9	41	0	0.0122	1.0000	0.0025	0.0025
5.0	5.0	195	0	0.0026	0.9974	0.0005	0.0005	4.9	4.9	41	0	0.0122	0.9878	0.0025	0.0025
10.0	5.0	195	0	0.0026	0.9949	0.0005	0.0005	9.8	4.9	41	0	0.0122	0.9758	0.0024	0.0025
15.0	5.0	195	0	0.0026	0.9923	0.0005	0.0005	14.7	4.9	41	0	0.0122	0.9639	0.0024	0.0025
20.0	5.0	195	5	0.0256	0.9898	0.0051	0.0052	19.7	4.9	41	1	0.0244	0.9521	0.0047	0.0050
25.0	5.0	190	52	0.2737	0.9644	0.0528	0.0635	24.6	4.9	40	12	0.3000	0.9289	0.0567	0.0718
30.0	5.0	138	14	0.1267	0.7005	0.0178	0.0271	29.5	4.9	28	3	0.1364	0.6502	0.0180	0.0298
35.0	5.0	69	0	0.0103	0.6117	0.0013	0.0021	34.4	4.9	13	0	0.0526	0.5616	0.0060	0.0110
40.0	5.0	28	0	0.0345	0.6054	0.0042	0.0070	39.3	4.9	6	0	0.1429	0.5320	0.0155	0.0313
45.0		1	0	1.0000	0.5845			44.2		1	0	1.0000	0.4560		
**UDDER DISEASES**								**LOCOMOTOR SYSTEM DISEASES**							
0.0	5.0	686	0	0.0007	1.0000	0.0001	0.0001	0.0	5.0	591	0	0.0008	1.0000	0.0002	0.0002
5.0	5.0	686	0	0.0007	0.9993	0.0001	0.0001	5.0	5.0	591	0	0.0008	0.9992	0.0002	0.0002
10.0	5.0	686	0	0.0007	0.9985	0.0001	0.0001	10.0	5.0	591	0	0.0008	0.9983	0.0002	0.0002
15.0	5.0	686	0	0.0007	0.9978	0.0001	0.0001	15.0	5.0	591	0	0.0008	0.9975	0.0002	0.0002
20.0	5.0	686	19	0.0277	0.9971	0.0055	0.0056	20.0	5.0	591	17	0.0288	0.9966	0.0057	0.0058
25.0	5.0	667	171	0.2564	0.9695	0.0498	0.0589	25.0	5.0	574	163	0.2840	0.9680	0.0550	0.0662
30.0	5.0	496	55	0.1338	0.7209	0.0193	0.0287	30.0	5.0	411	45	0.1400	0.6931	0.0194	0.0301
34.9	5.0	271	0	0.0027	0.6245	0.0003	0.0005	35.0	5.0	187	0	0.0040	0.5961	0.0005	0.0008
39.9	5.0	105	0	0.0094	0.6228	0.0012	0.0019	40.0	5.0	62	0	0.0159	0.5937	0.0019	0.0032
44.9		1	0	1.0000	0.6169			45.0		1	0	1.0000	0.5843		
**REPRODUCTIVE DISORDERS**								**ACCIDENTS**							
0.0	5.0	1878	0	0.0003	1.0000	0.0001	0.0001	0.0	5.0	577	0	0.0009	1.0000	0.0002	0.0002
5.0	5.0	1878	0	0.0003	0.9997	0.0001	0.0001	5.0	5.0	577	0	0.0009	0.9991	0.0002	0.0002
10.0	5.0	1878	0	0.0003	0.9995	0.0001	0.0001	10.0	5.0	577	0	0.0009	0.9983	0.0002	0.0002
15.0	5.0	1878	0	0.0003	0.9992	0.0001	0.0001	15.0	5.0	577	0	0.0009	0.9974	0.0002	0.0002

20.0	5.0	1878	4	0.0021	0.9989	0.0004	0.0004	20.0	5.0	577	20	0.0347	0.9965	0.0069	0.0071
25.0	5.0	1874	93	0.0496	0.9968	0.0099	0.0102	24.9	5.0	557	145	0.2603	0.9620	0.0502	0.0600
30.0	5.0	1781	39	0.0238	0.9473	0.0045	0.0048	29.9	5.0	412	33	0.0936	0.7116	0.0134	0.0197
35.0	5.0	1456	0	0.0005	0.9248	0.0001	0.0001	34.9	5.0	260	0	0.0029	0.6450	0.0004	0.0006
40.0	5.0	750	0	0.0013	0.9244	0.0002	0.0003	39.9	5.0	87	0	0.0114	0.6431	0.0015	0.0023
45.0		6	0	0.1667	0.9231			44.9		1	0	1.0000	0.6358		
**METABOLIC AND DIGESTIVE SYSTEM DISEASES**								**OTHER**							
0.0	5.0	435	0	0.0011	1.0000	0.0002	0.0002	0.0	5.0	501	0	0.0010	1.0000	0.0002	0.0002
5.0	5.0	435	0	0.0011	0.9989	0.0002	0.0002	5.0	5.0	501	0	0.0010	0.9990	0.0002	0.0002
10.0	5.0	435	0	0.0011	0.9977	0.0002	0.0002	10.0	5.0	501	0	0.0010	0.9980	0.0002	0.0002
15.0	5.0	435	0	0.0011	0.9966	0.0002	0.0002	15.0	5.0	501	0	0.0010	0.9970	0.0002	0.0002
20.0	5.0	435	17	0.0391	0.9954	0.0078	0.0080	20.0	5.0	501	17	0.0339	0.9960	0.0068	0.0069
25.0	5.0	418	143	0.3421	0.9565	0.0654	0.0825	25.0	5.0	484	116	0.2397	0.9622	0.0461	0.0545
30.0	5.0	275	38	0.1731	0.6293	0.0218	0.0379	30.0	5.0	368	26	0.0821	0.7316	0.0120	0.0171
35.0	5.0	126	0	0.0056	0.5203	0.0006	0.0011	35.0	5.0	239	0	0.0029	0.6715	0.0004	0.0006
40.0	5.0	51	0	0.0192	0.5174	0.0020	0.0039	40.0	5.0	106	0	0.0093	0.6696	0.0013	0.0019
45.0		1	0	1.0000	0.5075			45.0		1	0	1.0000	0.6633		

X_1—beginning of the time interval.
H_i—time interval width.
l_1—the number of cows entering the interval alive.
l_Z—the number of deaths.
PZ—the proportion of deaths = the number of deaths/the number of cases at risk in the interval.
Sp—the cumulative proportion of surviving cows until the time interval (the product of survival probabilities of all preceding intervals).
f_i—probability density function, i.e., the probability of culling in the time interval per unit time:
$f_i = \frac{P_i - P_{i+1}}{H_i}$, where P_i is the cumulative proportion of surviving cases at the beginning of the interval and the beginning of the subsequent interval (P_i+1), H_i is the time interval width.
h_i–hazard rate:
$h_i = \frac{f_i}{0{,}5 \cdot (P_i + P_{i+1})}$, where f_i—probability density function, P_i – P_i is the cumulative proportion of surviving cases at the beginning of the interval and the beginning of the subsequent interval (P_i+1).

Table S2. Survival tables for cows until the second lactation according to the culling categories.

X_1	H_i	l_1	L_Z	*PZ*	*Sp*	f_i	h_i	X_1	H_i	l_1	L_Z	*PZ*	*Sp*	f_i	h_i
**LOW MILK YIELD**								**RESPIRATORY SYSTEM DISEASES**							
0.0	7.8	427	0	0.0012	1.0000	0.0002	0.0002	0.0	7.1	87	0	0.0057	1.0000	0.0008	0.0008
7.8	7.8	427	0	0.0012	0.9988	0.0002	0.0002	7.1	7.1	87	0	0.0057	0.9943	0.0008	0.0008
15.5	7.8	427	1	0.0023	0.9977	0.0003	0.0003	14.1	7.1	87	0	0.0057	0.9885	0.0008	0.0008
23.3	7.8	426	70	0.1645	0.9953	0.0211	0.0231	21.2	7.1	87	9	0.1034	0.9829	0.0144	0.0154
31.1	7.8	355	22	0.0710	0.8316	0.0076	0.0095	28.3	7.1	78	8	0.1119	0.8812	0.0139	0.0168
38.9	7.8	243	75	0.3371	0.7726	0.0335	0.0522	35.4	7.1	57	8	0.1553	0.7826	0.0172	0.0238
46.6	7.8	127	75	0.5976	0.5121	0.0394	0.1097	42.4	7.1	38	22	0.5946	0.6610	0.0556	0.1197
54.4	7.8	49	31	0.6889	0.2061	0.0183	0.1352	49.5	7.1	14	10	0.7143	0.2680	0.0271	0.1572
62.2	7.8	10	0	0.0909	0.0641	0.0008	0.0123	56.6	7.1	4	2	0.5714	0.0766	0.0062	0.1131
69.9		1	0	1.0000	0.0583			63.6		1	0	1.0000	0.0328		
**UDDER DISEASES**								**LOCOMOTOR SYSTEM DISEASES**							
0.0	7.8	1862	0	0.0003	1.0000	0.0000	0.0000	0.0	7.8	1438	0	0.0003	1.0000	0.0000	0.0000
7.8	7.8	1862	0	0.0003	0.9997	0.0000	0.0000	7.8	7.8	1438	0	0.0003	0.9997	0.0000	0.0000
15.6	7.8	1862	4	0.0021	0.9995	0.0003	0.0003	15.7	7.8	1438	4	0.0028	0.9993	0.0004	0.0004

23.5	7.8	1858	241	0.1301	0.9973	0.0166	0.0178
31.3	7.8	1607	84	0.0575	0.8676	0.0064	0.0076
39.1	7.8	1233	437	0.3790	0.8178	0.0396	0.0598
46.9	7.8	636	409	0.6441	0.5078	0.0418	0.1215
54.7	7.8	225	130	0.6357	0.1807	0.0147	0.1192
62.5	7.8	54	0	0.0179	0.0658	0.0002	0.0023
70.4		2	0	0.5000	0.0647		
**REPRODUCTIVE DISORDERS**							
0.0	7.9	5090	0	0.0001	1.0000	0.0000	0.0000
7.9	7.9	5090	0	0.0001	0.9999	0.0000	0.0000
15.8	7.9	5090	2	0.0004	0.9998	0.0000	0.0000
23.7	7.9	5088	134	0.0264	0.9994	0.0033	0.0034
31.5	7.9	4928	50	0.0111	0.9730	0.0014	0.0014
39.4	7.9	4005	489	0.1407	0.9622	0.0172	0.0192
47.3	7.9	2456	1165	0.4743	0.8268	0.0497	0.0789
55.2	7.9	1291	620	0.5422	0.4346	0.0299	0.0943
63.1	7.9	376	0	0.0027	0.1990	0.0001	0.0003
71.0		1	0	1.0000	0.1984		
**CONTAGIOUS DISEASES**							
0.0	7.6	13	0	0.0385	1.0000	0.0051	0.0052
7.6	7.6	13	0	0.0385	0.9615	0.0049	0.0052
15.1	7.6	13	0	0.0385	0.9246	0.0047	0.0052
22.7	7.6	13	0	0.0385	0.8890	0.0045	0.0052
30.2	7.6	13	2	0.1667	0.8548	0.0188	0.0241
37.8	7.6	9	4	0.4444	0.7123	0.0419	0.0756
45.4	7.6	5	2	0.4000	0.3957	0.0209	0.0661
52.9	7.6	3	0	0.1667	0.2374	0.0052	0.0241
60.5	7.6	3	0	0.2500	0.1979	0.0065	0.0378
68.0		1	0	1.0000	0.1484		
**METABOLIC AND DIGESTIVE SYSTEM DISEASES**							
0.0	7.8	1085	0	0.0005	1.0000	0.0001	0.0001
7.8	7.8	1085	0	0.0005	0.9995	0.0001	0.0001
15.6	7.8	1085	3	0.0028	0.9991	0.0004	0.0004
23.4	7.8	1082	195	0.1808	0.9963	0.0231	0.0254
31.3	7.8	880	91	0.1145	0.8162	0.0120	0.0155
39.1	7.8	618	294	0.5052	0.7227	0.0467	0.0865
46.9	7.8	252	163	0.6481	0.3576	0.0297	0.1227
54.7	7.8	88	48	0.5854	0.1258	0.0094	0.1059
62.5	7.8	28	0	0.0345	0.0522	0.0002	0.0045
70.3		1	0	1.0000	0.0504		

23.5	7.8	1434	221	0.1547	0.9965	0.0197	0.0214
31.3	7.8	1203	53	0.0499	0.8424	0.0054	0.0065
39.1	7.8	869	316	0.3837	0.8004	0.0392	0.0607
47.0	7.8	462	278	0.6024	0.4933	0.0380	0.1101
54.8	7.8	183	100	0.6024	0.1961	0.0151	0.1102
62.6	7.8	49	0	0.0200	0.0780	0.0002	0.0026
70.4		1	0	1.0000	0.0764		
**ACCIDENTS**							
0.0	7.9	1360	0	0.0004	1.0000	0.0000	0.0000
7.9	7.9	1360	0	0.0004	0.9996	0.0000	0.0000
15.7	7.9	1360	5	0.0037	0.9993	0.0005	0.0005
23.6	7.9	1355	194	0.1440	0.9956	0.0182	0.0197
31.4	7.9	1146	78	0.0764	0.8523	0.0083	0.0101
39.3	7.9	817	256	0.3434	0.7871	0.0344	0.0527
47.2	7.9	418	228	0.5455	0.5168	0.0359	0.0954
55.0	7.9	190	106	0.6310	0.2349	0.0189	0.1173
62.9	7.9	40	0	0.0244	0.0867	0.0003	0.0031
70.7		1	0	1.0000	0.0846		
**OTHER**							
0.0	7.9	1215	0	0.0004	1.0000	0.0001	0.0001
7.9	7.9	1215	0	0.0004	0.9996	0.0001	0.0001
15.7	7.9	1215	5	0.0041	0.9992	0.0005	0.0005
23.6	7.9	1210	154	0.1282	0.9951	0.0162	0.0174
31.5	7.9	1039	69	0.0735	0.8675	0.0081	0.0097
39.3	7.9	770	227	0.3276	0.8038	0.0335	0.0498
47.2	7.9	389	226	0.5810	0.5405	0.0399	0.1041
55.0	7.9	163	78	0.5342	0.2265	0.0154	0.0927
62.9	7.9	51	0	0.0192	0.1055	0.0003	0.0025
70.8		1	0	1.0000	0.1035		

X_1—beginning of the time interval.

H_i—time interval width.

l_1—the number of cows entering the interval alive.

l_Z—the number of deaths.

PZ—the proportion of deaths = the number of deaths/the number of cases at risk in the interval.

Sp—the cumulative proportion of surviving cows until the time interval (the product of survival probabilities of all preceding intervals).

f_i—probability density function, i.e., the probability of culling in the time interval per unit time:

$f_i = \frac{P_i - P_{i+1}}{H_i}$, where P_i is the cumulative proportion of surviving cases at the beginning of the interval and the beginning of the subsequent interval (P_i+1), H_i is the time interval width.

h_i–hazard rate:

$h_i = \frac{f_i}{0,5\cdot(P_i+P_{i+1})}$, where f_i—probability density function, P_i – P_i is the cumulative proportion of surviving cases at the beginning of the interval and the beginning of the subsequent interval (P_i+1).

Table S3. Survival tables for cows until the third lactation according to the culling categories.

X_1	H_i	l_1	L_Z	PZ	Sp	f_i	h_i	X_1	H_i	l_1	L_Z	PZ	Sp	f_i	h_i
LOW MILK YIELD								RESPIRATORY SYSTEM DISEASES							
0.0	10.5	608	0	0.0008	1.0000	0.0001	0.0001	0.0	10.2	126	0	0.0040	1.0000	0.0004	0.0004
10.5	10.5	608	0	0.0008	0.9992	0.0001	0.0001	10.2	10.2	126	0	0.0040	0.9960	0.0004	0.0004
21.1	10.5	608	71	0.1176	0.9984	0.0112	0.0119	20.3	10.2	126	13	0.1032	0.9921	0.0101	0.0107
31.6	10.5	528	43	0.0902	0.8809	0.0075	0.0090	30.5	10.2	113	11	0.1078	0.8897	0.0094	0.0112
42.1	10.5	382	133	0.3590	0.8014	0.0273	0.0415	40.7	10.2	80	28	0.3590	0.7938	0.0280	0.0430
52.6	10.5	226	123	0.5566	0.5137	0.0272	0.0732	50.9	10.2	48	27	0.5625	0.5088	0.0281	0.0769
63.2	10.5	93	59	0.6821	0.2278	0.0148	0.0983	61.0	10.2	21	15	0.7500	0.2226	0.0164	0.1179
73.7	10.5	21	0	0.0357	0.0724	0.0002	0.0035	71.2	10.2	4	0	0.2000	0.0557	0.0011	0.0218
84.2	10.5	7	0	0.1250	0.0698	0.0008	0.0127	81.4	10.2	1	0	0.5000	0.0445	0.0022	0.0655
94.8		1	0	1.0000	0.0611			91.6		1	0	1.0000	0.0223		
UDDER DISEASES								LOCOMOTOR SYSTEM DISEASES							
0.0	10.5	2990	0	0.0002	1.0000	0.0000	0.0000	0.0	10.5	2250	0	0.0002	1.0000	0.0000	0.0000
10.5	10.5	2990	0	0.0002	0.9998	0.0000	0.0000	10.5	10.5	2250	0	0.0002	0.9998	0.0000	0.0000
21.0	10.5	2990	245	0.0822	0.9997	0.0078	0.0082	21.1	10.5	2250	225	0.1005	0.9996	0.0095	0.0100
31.5	10.5	2726	209	0.0821	0.9175	0.0072	0.0081	31.6	10.5	2003	154	0.0835	0.8991	0.0071	0.0083
42.0	10.5	2157	714	0.3375	0.8422	0.0271	0.0386	42.1	10.5	1532	496	0.3285	0.8240	0.0257	0.0373
52.5	10.5	1360	742	0.5548	0.5579	0.0295	0.0731	52.6	10.5	992	523	0.5389	0.5534	0.0283	0.0701
63.0	10.5	573	313	0.5968	0.2484	0.0141	0.0809	63.2	10.5	426	237	0.6054	0.2552	0.0147	0.0824
73.6	10.5	163	0	0.0048	0.1002	0.0000	0.0005	73.7	10.5	120	0	0.0061	0.1007	0.0001	0.0006
84.1	10.5	47	0	0.0204	0.0997	0.0002	0.0020	84.2	10.5	44	0	0.0222	0.1001	0.0002	0.0021
94.6		2	0	0.5000	0.0977			94.8		1	0	1.0000	0.0979		
REPRODUCTIVE DISORDERS								ACCIDENTS							
0.0	10.5	7741	0	0.0001	1.0000	0.0000	0.0000	0.0	10.5	2109	0	0.0002	1.0000	0.0000	0.0000
10.5	10.5	7741	0	0.0001	0.9999	0.0000	0.0000	10.5	10.5	2109	0	0.0002	0.9998	0.0000	0.0000
21.1	10.5	7741	136	0.0176	0.9999	0.0017	0.0017	21.0	10.5	2109	199	0.0948	0.9995	0.0090	0.0095
31.6	10.5	7569	140	0.0203	0.9823	0.0019	0.0019	31.6	10.5	1892	163	0.0938	0.9048	0.0081	0.0094
42.2	10.5	6101	1248	0.2150	0.9623	0.0196	0.0228	42.1	10.5	1420	442	0.3205	0.8199	0.0250	0.0363
52.7	10.5	4258	1647	0.4009	0.7554	0.0287	0.0475	52.6	10.5	896	465	0.5330	0.5571	0.0282	0.0691
63.3	10.5	2312	862	0.4280	0.4526	0.0184	0.0516	63.1	10.5	384	202	0.5714	0.2602	0.0141	0.0761
73.8	10.5	854	0	0.0009	0.2589	0.0000	0.0001	73.6	10.5	121	0	0.0063	0.1115	0.0001	0.0006
84.4	10.5	252	0	0.0039	0.2586	0.0001	0.0004	84.1	10.5	38	0	0.0256	0.1108	0.0003	0.0025
94.9		6	0	0.1667	0.2576			94.7		1	0	1.0000	0.1080		
CONTAGIOUS DISEASES								OTHER							
0.0	9.9	30	0	0.0167	1.0000	0.0017	0.0017	0.0	10.5	1870	0	0.0003	1.0000	0.0000	0.0000
9.9	9.9	30	0	0.0167	0.9833	0.0017	0.0017	10.5	10.5	1870	0	0.0003	0.9997	0.0000	0.0000
19.7	9.9	30	0	0.0167	0.9669	0.0016	0.0017	21.1	10.5	1870	159	0.0855	0.9995	0.0081	0.0085
29.6	9.9	30	3	0.1034	0.9508	0.0100	0.0111	31.6	10.5	1690	143	0.0919	0.9140	0.0080	0.0091
39.4	9.9	25	5	0.2000	0.8525	0.0173	0.0226	42.2	10.5	1280	389	0.3141	0.8300	0.0247	0.0353
49.3	9.9	20	3	0.1500	0.6820	0.0104	0.0165	52.7	10.5	808	415	0.5260	0.5693	0.0284	0.0677
59.1	9.9	17	5	0.3226	0.5797	0.0190	0.0390	63.3	10.5	355	173	0.5406	0.2699	0.0138	0.0702
69.0	9.9	9	1	0.1429	0.3927	0.0057	0.0156	73.8	10.5	112	0	0.0068	0.1240	0.0001	0.0006
78.8	9.9	4	0	0.2000	0.3366	0.0068	0.0226	84.4	10.5	36	0	0.0270	0.1231	0.0003	0.0026
88.7		1	0	1.0000	0.2693			94.9		1	0	1.0000	0.1198		

**METABOLIC AND DIGESTIVE SYSTEM DISEASES**							
0.0	10.5	1746	0	0.0003	1.0000	0.0000	0.0000
10.5	10.5	1746	0	0.0003	0.9997	0.0000	0.0000
21.0	10.5	1746	198	0.1141	0.9994	0.0108	0.0115
31.6	10.5	1528	203	0.1417	0.8854	0.0119	0.0145
42.1	10.5	1135	439	0.3939	0.7600	0.0285	0.0466
52.6	10.5	655	416	0.6415	0.4606	0.0281	0.0898
63.1	10.5	226	138	0.6651	0.1651	0.0104	0.0947
73.7	10.5	51	0	0.0154	0.0553	0.0001	0.0015
84.2	10.5	14	0	0.0625	0.0545	0.0003	0.0061
94.7		2	0	0.5000	0.0511		

X_1—beginning of the time interval.

H_i—time interval width.

l_1—the number of cows entering the interval alive.

l_Z—the number of deaths.

PZ—the proportion of deaths = the number of deaths/the number of cases at risk in the interval.

Sp—the cumulative proportion of surviving cows until the time interval (the product of survival probabilities of all preceding intervals).

f_i—probability density function, i.e., the probability of culling in the time interval per unit time:

$f_i = \frac{P_i - P_{i+1}}{H_i}$, where P_i is the cumulative proportion of surviving cases at the beginning of the interval and the beginning of the subsequent interval (P_i+1), H_i is the time interval width.

h_i–hazard rate:

$h_i = \frac{f_i}{0,5 \cdot (P_i + P_{i+1})}$, where f_i—probability density function, P_i – P_i is the cumulative proportion of surviving cases at the beginning of the interval and the beginning of the subsequent interval (P_i+1).
